# Cardiac Changes in Parkinson’s Disease: Lessons from Clinical and Experimental Evidence

**DOI:** 10.3390/ijms222413488

**Published:** 2021-12-16

**Authors:** Lorena Cuenca-Bermejo, Pilar Almela, Javier Navarro-Zaragoza, Emiliano Fernández Villalba, Ana-María González-Cuello, María-Luisa Laorden, María-Trinidad Herrero

**Affiliations:** 1 Clinical and Experimental Neuroscience Group/Biomedical Research Institute of Murcia (NiCE-IMIB)/Institute for Aging Research, School of Medicine, University of Murcia, 30100 Murcia, Spain; lorena.cuenca@um.es (L.C.-B.); agcuello@um.es (A.-M.G.-C.); 2 Department of Pharmacology, School of Medicine, Biomedical Research Institute of Murcia (IMIB), University of Murcia, 30100 Murcia, Spain; palmela@um.es (P.A.); jnavarrozaragoza@um.es (J.N.-Z.); laorden@um.es (M.-L.L.)

**Keywords:** Parkinson’s disease, neurodegeneration, cardiac denervation, autonomic nervous system, L-DOPA, dysautonomia, aging

## Abstract

Dysautonomia is a common non-motor symptom in Parkinson’s disease (PD). Most dysautonomic symptoms appear due to alterations in the peripheral nerves of the autonomic nervous system, including both the sympathetic and parasympathetic nervous systems. The degeneration of sympathetic nerve fibers and neurons leads to cardiovascular dysfunction, which is highly prevalent in PD patients. Cardiac alterations such as orthostatic hypotension, heart rate variability, modifications in cardiogram parameters and baroreflex dysfunction can appear in both the early and late stages of PD, worsening as the disease progresses. In PD patients it is generally found that parasympathetic activity is decreased, while sympathetic activity is increased. This situation gives rise to an imbalance of both tonicities which might, in turn, promote a higher risk of cardiac damage through tachycardia and vasoconstriction. Cardiovascular abnormalities can also appear as a side effect of PD treatment: L-DOPA can decrease blood pressure and aggravate orthostatic hypotension as a result of a negative inotropic effect on the heart. This unwanted side effect limits the therapeutic use of L-DOPA in geriatric patients with PD and can contribute to the number of hospital admissions. Therefore, it is essential to define the cardiac features related to PD for the monitorization of the heart condition in parkinsonian individuals. This information can allow the application of intervention strategies to improve the course of the disease and the proposition of new alternatives for its treatment to eliminate or reverse the motor and non-motor symptoms, especially in geriatric patients.

## 1. Introduction

Parkinson’s disease (PD) is broadly known to be a movement disorder, originating from the reduction of brain dopamine (DA) content as a consequence of the degeneration of the nigrostriatal system, together with the presence of proteinaceous cytoplasmic inclusions enriched in α-synuclein, named Lewy bodies [1]. Developed more than 50 years ago, this definition based on the motor condition is no longer an accurate description for PD; numerous works have evidenced that clinical manifestations of the disease go beyond the motor system and set up a complex scenario in which non-motor symptoms appear even years before the motor ones [2,3]. Nowadays, it is known that the complete spectrum of PD symptoms not only derives from the degeneration of the nigrostriatal system, but because other central nervous system (CNS) nuclei are also damaged, inducing the appearance of dysautonomia (alterations in the autonomic nervous system) [4]. Non-motor symptoms derived from autonomic dysfunction include, among others, urinary problems, constipation, erectile failure in men, orthostatic intolerance or orthostatic hypotension (OH) [5,6]. Therefore, it seems clear that the study of CNS alterations together with systemic changes would provide a better understanding of the disease, in terms of its progression, and the development of diagnostic tools and therapeutical strategies [7].

Increasing evidence highlights that cardiovascular impairment is an important non-motor sign in the prodromal phases of PD and it worsens as the disease progresses [8,9]. In fact, PD and cardiovascular diseases have common risk factors: oxidative stress, maintained inflammatory processes, diabetes, obesity and hypertension [10,11,12]. Importantly, both conditions are comorbidities that appear during aging. Knowing that cardiovascular diseases are one of the main causes of death in the world and that the incidence and prevalence of neurodegenerative disorders is continuously increasing, it is important to explore their relationship in order to understand how each condition affects the other, and vice versa.

Different cardiac alterations (due to autonomic dysfunction) have been detected in PD patients [13]. Thus, the specific concept of “the Parkinsonian Heart” has become more popular during the last decades, since PD patients show unique cardiac features that are different from age-matched control subjects and also from other forms of systemic dysfunction [14]. These alterations of the cardiovascular system include sympathetic denervation, functional and structural modifications, and changes at the molecular level [15]. In addition, parasympathetic dysfunction in PD patients has been associated with the outcome of OH. Therefore, it is suggested that both cardiac sympathetic denervation and parasympathetic dysfunction can occur concurrently [16]. Cardiovascular disorders have been detected in approximately 80% of PD patients [17], with OH being the most common cardiac-related autonomic dysfunction (30–40% of cases) [15]. Investigating the cardiac condition in PD could provide a more accurate (and earlier) in vivo diagnosis. On the other hand, establishing the features of the “Parkinsonian Heart” allows monitoring of the patients to prevent undesirable consequences of heart failure, as well as the adaptation of precise treatments.

This work reviews the relationship between cardiac alterations and PD, with a special focus on clinical findings (including the effect of antiparkinsonian treatments) and the evidence provided by experimental models used in PD research.

## 2. Cardiac Sympathetic Loss in PD

Numerous studies have provided evidence that PD not only features neurodegeneration in the dopaminergic system, but other brain areas are also known to be affected, such as the pedunculopontine nucleus, the locus coeruleus (LC), the rostro ventrolateral medulla (RVLM), and the nucleus of the tractus solitarius (NTS) [18,19,20,21]. In particular, a marked loss of noradrenaline (NA) has been demonstrated in the early stages, that advances within the progression of the disease [22,23].

NA is a catecholaminergic neurotransmitter in the sympathetic nervous system, which stimulates adrenergic receptors (PubChem CID: 439260). NA is the main neurotransmitter of the majority of postganglionic sympathetic fibers and of the brain projection system coming from the LC. In the heart, activation of β-1 adrenergic receptors produces an increase in myocardial contractility, heart rate, and atrioventricular conduction; on the contrary, the stimulation of β-2 adrenergic receptors induces vascular smooth muscle dilation.

**Figure 1:**
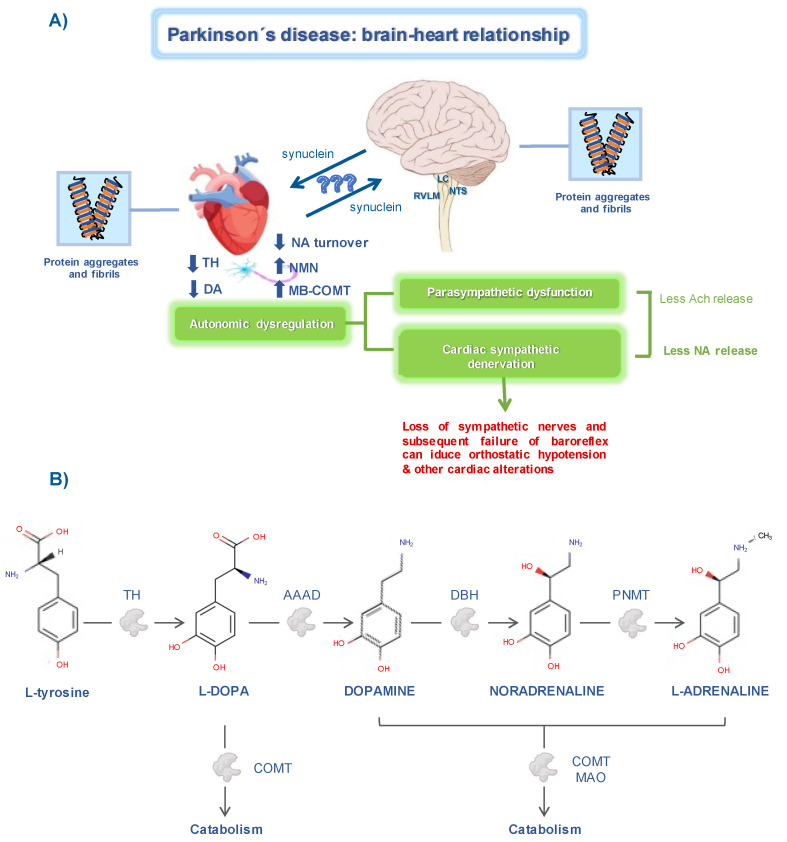
Brain–heart relationship in Parkinson’s disease. (**A**) Changes in cardiac sympathetic pathways. The decrease in tyrosine hydroxylase (TH) either at the brain or cardiac level, induces alterations in noradrenaline (NA) metabolism. In PD patients, alpha-synuclein aggregates can be found in the myocardial tissue and in cardiac-related brain regions, such as the locus coeruleus (LC), nucleus of the tractus solitarius (NTS), and the rostro ventrolateral medulla (RVLM). Altogether, these abnormalities in the autonomic system are related to the development of cardiac dysfunction. (**B**) Schematic representation of the catecholamines’ biosynthetic pathway. Tyrosine hydroxylase (TH), aromatic amino acid decarboxylase (AAAD), dopamine-β-hydroxylase (DBH), phenylethanolamine-N-methyltransferase (PNMT), catechol-O-methyltransferase (COMT), monoamine oxidase (MAO). Chemical structures were obtained from the DrugBank database.

After years of research, we currently know that noradrenergic degeneration in the CNS is as important as dopaminergic degeneration [24]. This knowledge, together with the appearance of autonomic dysfunction, inspired the thought that noradrenergic innervation could also be altered outside the CNS. This relationship is supported by the fact that most of the CNS regions affected in PD are in charge of regulating the activity of the autonomic nervous system, and their degeneration is coupled with the specific reduction of sympathetic terminals in the heart (Figure 1A) [14]. In addition, DA and NA share their biosynthetic pathway, which is damaged in PD (Figure 1B).

The origin of sympathetic failure in PD is a controversial topic: is it the result of the degeneration of CNS neurons, or is it due to the loss of peripheral sympathetic fibers? The available studies point towards a complex and variable scenario. On the one hand, α-synuclein accumulation in the lower brain stem and spinal cord has been found in the very early stages of the disease, including in the autonomic pontine nuclei (such as the LC) and the sympathetic autonomic nuclei (such as the RVLM and NTS) [18,25]. Altogether, these findings suggest that degeneration of both parasympathetic and sympathetic preganglionic neurons in the CNS occurs in PD. Studies evaluating the parasympathetic impairment support this idea: PD patients presenting with autonomic failure show a lack of peripheral α-synuclein pathology and there is no evidence of peripheral sympathetic nerve degeneration, thus indicating that sympathetic denervation must originate in the CNS [8,26]. On the contrary, numerous authors suggest that peripheral sympathetic degeneration starts even before brain pathology is advanced, and this is supported by the fact that α-synuclein deposits have been found in peripheral tissues [27,28]. Nowadays, most studies suggest that the autonomic dysfunction might appear in an independent way of the dopaminergic cell loss, which is mainly responsible for rigidity and bradykinesia of motor symptoms in PD [15].

The sympathetic system controls increases in heart rate, while the parasympathetic one is in charge of its decrease [29]. Different evidence points out that in the early phases of PD, there might be a decrease in parasympathetic activity and an increment in sympathetic activity, resulting in the dysregulation of the autonomous system [30]. On the other hand, different works have shown that there is a specific sympathetic loss in the cardiac tissue of PD patients, which precedes clinical motor symptoms of PD [13,31].

In summary, it is nowadays accepted that the pathological picture of PD, such as catecholaminergic dysfunction, is not confined to the brain, but also includes peripheral structures.

### 2.1. Neuroimaging Findings

The current methodology to clinically evaluate the loss of noradrenergic innervation in the heart is mainly based on the use of peripheral tracers that bind to catecholaminergic structures [14]: (i) positron emission tomography (PET) with 6-[^18^F]fluorodopamine (6-[^18^F]DA, PubChem CID: 450112) [32,33]; and (ii) scintigraphy, single photon emission computed tomography (SPECT) and PET with the NA analog ^123^I-metaiodobenzyl-guanidine (MIBG, PubChem CID: 450504) [34,35,36,37,38]. The determination of NA plasma levels and cardiac turnover of its metabolites are also used [13,32,34]. In addition, tyrosine hydroxylase (TH, UniProtKB ID: P07101) immunodetection performed on *postmortem* myocardial tissue is accepted to study cardiac sympathetic denervation [39,40].

An increasing number of PET scan studies using MIBG and 6-[^18^F]DA have shown that most PD patients have low radioactivity concentrations when the cardiac retention of these agents is explored, which translates into a loss of sympathetic innervation in the heart [13,14,16]. For this reason, in 2015 the Movement Disorder Society approved the presence of sympathetic denervation in the heart (by means of cardiac MIBG scintigraphy) as a valid criterion for the clinical diagnosis of PD [41]. This clinical evidence has been subsequently correlated with *postmortem* findings: there is a reduction of the TH-immunoreactive (TH-ir) fibers in the cardiac tissue of PD patients with autonomic failure [40,42]. Moreover, the loss of sympathetic innervation seems to follow a pattern: its decrease is more pronounced in the myocardium of the left ventricle and, while it is relatively preserved in the septum or in the anterior wall, most patients have the denervation located in the inferior or lateral walls [33,43].

Histological analyses with hematoxylin-eosin staining have revealed that there are no abnormalities in the cardiac nerve bundles of PD patients presenting with autonomic failure [40]. These findings are supported by the study conducted by Krämer and collaborators, who concluded that sympathetic impairment observed in PD is derived from CNS degeneration more than from peripheral nerve fiber destruction [8]. Therefore, the autonomic impairment is the result of the loss of sympathetic innervation but not actual nerve degeneration [44]. Importantly, α-synuclein accumulation has been detected in myocardial tissue and in coronary arteries in some PD cases with cardiac sympathetic alterations [45,46].

Putting all the evidence together, the sympathetic loss in the heart of PD patients has been described to affect the ventricles, atria, and electrical conduction system [13,33,42,46]. The selective sympathetic cardiac denervation can be found both in the early and in the late stages of the disease [47]. Interestingly, some studies have reported that PD subjects that showed little or no decrease in the MIBG or 6-[^18^F]DA signal, advanced likewise to a marked reduction of these sympathoneural tracers in the following years as the disease progressed, especially in the lateral ventricular wall [33]. These findings demonstrate that both the CNS and the autonomic nervous system are involved in PD pathology. In particular, what characterizes PD in this sense is a postglanglionic lesion, in which the loss might be found in axonal terminals despite the neuronal cell bodies being unaffected [4,36,48].

Chronic autonomic failure is not only a feature of PD. Some clinicians face the problem in making a clear diagnosis for PD, multiple system atrophy (MSA) and pure autonomic failure (PAF). However, recent studies have elucidated some characteristics that might be helpful to distinguish them. MSA is caused by a preganglionic degeneration (evident central neurodegeneration), the opposite to PD [49,50]. Several authors have demonstrated that scintigraphy with MIBG and PET scans using 6-[^18^F]DA can distinguish PD from MSA: whereas radioactivity is decreased in the heart of PD (and it progresses over time), in MSA it appears comparable to control subjects [9,51,52]. This means that in MSA, even in the cases that present OH, cardiac sympathetic innervation is intact [53]. Moreover, while PD patients show improved movement symptoms in response to L-DOPA treatment, people suffering from MSA do not respond to this therapy [4]. On the other hand, although the lesion found in PAF is usually postganglionic, such as in PD, NA plasma levels in PAF are lower than in PD [40,54,55].

Finally, although it has received less attention, cardiac parasympathetic dysfunction is also found in PD [16].

### 2.2. Circulating Catecholamine Levels

Dysfunction of the autonomic system is well stablished in PD. However, the etiology of PD-associated OH is complicated. Changing from the supine to the standing position is associated with a redistribution of blood (approximately 1 L) from the capacitance vessels of the inferior members and the splanchnic or pelvic circulation. As a result, venous return and cardiac output are reduced. In physiological conditions, the baroreflex counteracts this response and elevates the sympathetic outflow and promotes vagal inhibition. Then, heart rate, cardiac contractility as well as peripheral vascular resistance are elevated in order to maintain blood pressure [56]. An increase in NA plasma levels is needed to change from a supine to a standing position. OH is defined by the inability to compensate for a sudden systemic blood pressure decrease due to the loss of NA innervation [14,29].

NA release from the postganglionic sympathetic nerves has been shown to be decreased in patients with autonomic impairment, therefore making this the origin of the insufficient peripheral vasoconstriction in the arteries and OH [57]. This process may be associated with compensatory mechanisms governed by different neuroendocrine systems, such as vasopressin and adrenaline [56]. PD patients with OH (PD + OH) are not able to produce this increase in blood NA concentrations, which seem to be lower compared with PD patients without OH [58,59,60,61,62]. It is noteworthy, that NA circulating concentrations in PD + OH patients are lower, but no significant differences are found when they are compared with age-matched control subjects, just during the change from a supine to standing position [63]. This situation might be explained by two facts: the first one is that cardiac sympathetic denervation is partial, so the NA release might experience compensatory mechanisms in the remaining fibers; the second one is that measuring NA blood levels can fail to detect the real decrease in its liberation, since denervation also affects its reuptake [4].

In addition, it was found that NA levels were lowered in parkinsonian patients treated with L-DOPA or another dopaminergic agonist for more than one year. These individuals showed a maintained response to the therapy, while untreated PD patients did not show any changes when they were compared with control subjects. This fact could be explained knowing that TH decreases within the course of the disease [64].

## 3. Clinical Manifestations

Cardiovascular alterations can affect approximately 80% of PD patients and can worsen the progression of the disease, increasing the risk of death [17,29,65]. The most frequent clinical manifestation of cardiac autonomic failure in PD is OH [66].

**Figure 2:**
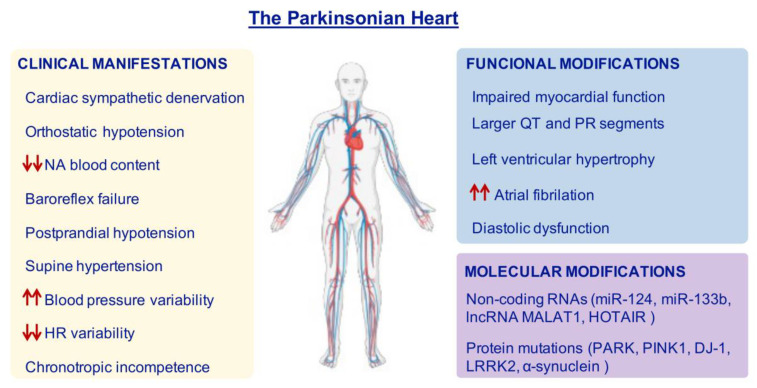
Features of the Parkinsonian Heart. Most PD patients can show cardiac alterations which reflect in clinical manifestations, functional and molecular modifications. Red arrows represent increase (up arrows) or decrease (down arrows). HR = heart rate; NA = noradrenaline.

All PD patients with OH have cardiac denervation [32]. However, even if OH can result from the specific loss of noradrenergic innervation in the heart, more factors must be involved since OH also appears in MSA, where no cardiac denervation is detected [32,49,67]. Altered baroreflex function has been described in PD patients and it has been related to OH [4]. Baroreflex failure in PD is characterized by a decreased sensitivity and involves both cardio-vagal and sympatho-neural circuits [62,68,69] (Figure 2), although more studies are needed to determine which pathological mechanisms are responsible for it.

Other cardiac alterations have also been found in PD patients with a loss of sympathetic innervation in the heart. For example, postprandial hypotension, supine hypertension, increased blood pressure variability and decreased heart rate variability, as well as chronotropic incompetence [63,70,71,72,73]. In fact, some authors support the idea that they could be used as early diagnostic tools, since some of them appear in the prodromal stages of the disease [29,70]. In particular, postprandial hypotension can appear in early stages of the disease and it is related to a worse outcome of the motor condition. Blood pressure falls and postprandial hypotension are more prevalent in PD patients presenting OH [74,75].

Heart failure is one of the leading causes of death among PD patients, with double the prevalence compared with the overall population [76,77]. For this reason, it is considered as a strong tool to predict mortality in PD patients [78].

On the other hand, PD is also associated with structural and functional modifications in the heart, which are found to be more severe in advanced stages [79]. Some works have found that PD patients have larger QT and PR segments compared to controls [80], although others have not [79].

Findings derived from echocardiographic studies have shown that PD is significantly associated with an increase in concentric left ventricular hypertrophy and diastolic (but not systolic) dysfunction. In particular, it has been shown that PD patients had a higher left atrial volume compared with the control group and a higher risk of atrial fibrillation [79,80,81,82]. Additionally, the evaluation of myocardial function has revealed that, in general, it worsens in PD subjects and specially as the disease progresses [82]. Altogether, these results may be related to the increased prevalence of heart failure in PD [29,79,83] (Figure 2). An open debate exists regarding the origin of abnormalities in the electrocardiographs of PD subjects: some studies point out that they might be caused as a consequence of some parkinsonian treatments, while others have found no relationship [79,80,83].

Heart failure is a comorbidity of natural aging, and aging is a risk factor to develop PD [30]. Therefore, the simultaneous presence of these conditions can worsen the progression of both of them. On the other hand, some authors have not found a positive correlation between the development of cardiac diseases and PD [84].

In PD patients, sudden death (SUDPAR) has been reported and it has been occasionally related to cardiac failure [85,86]. However, there is no consistent evidence that provides a unified definition for SUDPAR. Hence, cardiac function must be surveilled in PD patients, independently if it is a direct consequence of the disease or an age-associated comorbidity.

## 4. Molecular Alterations in the Parkinsonian Heart

Few studies in PD patients have focused on finding molecular keys to understand cardiac dysfunction. Beyond the pure nervous brain–heart connection, PD-related genes are also expressed in the heart, such as Parkin (PARK2), PINK1 (PARK6), DJ-1 (PARK7), LRRK2 (PARK8), and also α-synuclein (PARK1) [87,88,89]. Although it has not been possible to demonstrate a relationship between cardiac damage and Parkin deficiency in PD patients [90], several recent animal studies have suggested that Parkin protects from cardiac damage [91,92,93] (Figure 2).

PINK1 (UniProtKB ID: Q9BXM7) is a serine/threonine protein kinase and it has a key role in normal heart function, regulating mitochondrial dysfunction during stress responses [94,95,96]. In fact, KO mice for PINK1 develop cardiac hypertrophy associated with aging, resulting from mitochondrial dysfunction, impaired ATP production and enhanced oxidative stress [97]. When acute ischemic reperfusion injury is induced in this strain, it appears to be more susceptible to severe damage, and this has been associated with mitophagy defects [98]; however, there are no studies on its role as a cardioprotective agent in PD patients at the moment.

The physiological function of DJ-1 (UniProtKB ID: Q99497) is not clear, but it is described to be important during the oxidative stress response [99,100]. Its depletion on experimental models has been related to a higher susceptibility to cardiac damage in ischemia-reperfusion injury [97,101,102], ischemic preconditioning [103], and aortic constriction [104]. Thus, DJ-1 is also involved in the response to pathological stress in the heart [88].

The serine/threonine protein kinase LRRK2 (UniProtKB ID: Q5S007) participates in a broad range of pathways, but studies that focus on the analysis of cardiac performance in PD patients carrying mutations in this protein are scarce. Carricarte-Naranjo and collaborators (2019) performed an analysis to determine the possible role of LRRK2 mutations in cardiac manifestations in PD. They found that the LRRK2-G2019S mutation was significantly associated with an increase in global heart rate variability and beat-to-beat measures when the PD patients carrying the mutation were compared with both control subjects and PD patients with idiopathic origin [105].

Interestingly, the presence of aberrant α-synuclein (UniProtKB ID: P37840) aggregates has been detected in the epicardial tissue in non-diagnosed PD patients, leading the authors to theorize that this can be associated with a prodromal stage of the disease [106]. Importantly, Lewy bodies have been found in the myocardium of PD patients, particularly in the nerve fibers of the arteries and in the atrial ganglia [45,46].

Since PD patients show a higher risk of death from ischemic heart disease, mutations in PD-associated genes, specifically in Parkin (UniProtKB ID: O60260) and PINK1, should be considered for evaluation to clarify which mutations or genetic polymorphisms may also be related to cardiovascular disease [107]. Thus, future research might focus on the potential function of these proteins in the heart and use their mechanisms as means of cardioprotection.

During the last decades, the importance of noncoding RNAs (ncRNA) has increased due to their diverse biological implications. Several ncRNAs have been identified to have a possible role in the brain–heart axis, such as the miR-124 (OMIM ID: 609327), the miR-133b (OMIM ID: 610946), the lncRNA MALAT1 (OMIM ID: 607924), and the HOTAIR (OMIM ID: 611400) [108,109,110,111,112] (Figure 2). For an extended review, see reference [113].

## 5. Effects of Antiparkinsonian Treatments on the Heart

The effect of antiparkinsonian treatments is a source of controversy regarding their side effects: different authors have evaluated cardiac function in PD patients undergoing therapy and they have reached different conclusions, while some of them have found a negative relationship, others have not [114,115].

**Figure 3:**
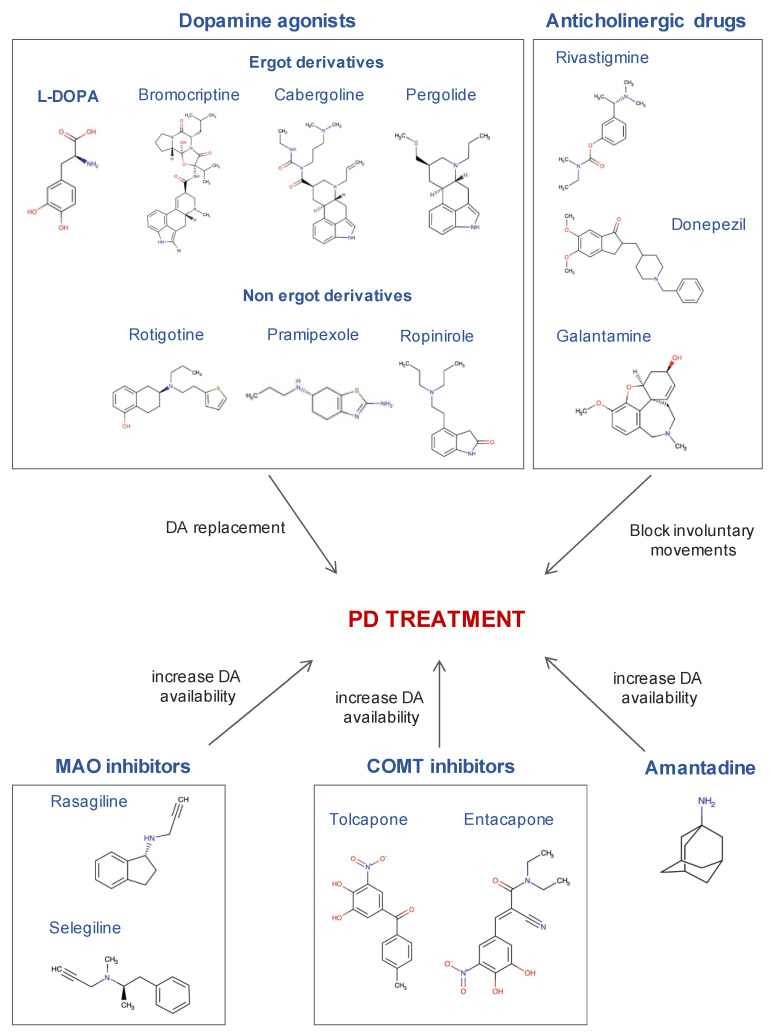
The most commonly used drugs in the treatment of Parkinson’s disease and their mechanism of action. Chemical structures were obtained from the DrugBank database.

The main available drugs for the classic treatment of PD include: L-DOPA, dopamine agonists, monoaminooxidase (MAO) B inhibitors, catechol ortho-methyltransferase (COMT) inhibitors, anticholinergic agents and amantadine. Unfortunately, most of these drugs can produce cardiac adverse effects, mainly in elderly patients and/or with previous pathologies (Figure 3).

Levodopa or L-DOPA, is a DA precursor and it is the gold standard for PD treatment (DrugBank ID: DB01235). L-DOPA can be converted to DA by DOPA decarboxylase on both sides of the blood brain barrier. Therefore, in the brain it compensates for the depleted DA levels of PD patients.

The possible detrimental effects of L-DOPA treatment on the cardiovascular system have been studied for years [116]. It has been proposed that L-DOPA intake is related to aortic stiffness, diastolic function, arterial pressure and cardiac contractility [114,117]. On the contrary, other authors have found that these alterations are independent of L-DOPA treatment [79]. In addition, it was shown that parkinsonian monkeys treated with L-DOPA had an increased NA turnover in the heart [118].

Since L-DOPA can induce unwanted side effects, reverse engineered drugs have been designed using L-DOPA as the starting molecule. In this line, the agonists for DA receptors in the brain have also been used as a strategy for PD treatment, mainly drugs with ergot-based structures. Among them: the D2 receptor agonists bromocriptine (DrugBank ID: DB01200) and cabergoline (DrugBank ID: DB00248), and the long-acting DA agonist pergolide (DrugBank ID: DB01186). Unfortunately, these drugs have been related to higher cardiovascular impairment in PD, such as fibrotic reactions in the heart and valvulopathies [119,120,121,122,123]. Subsequent studies have demonstrated that the toxicity of these drugs is not directly driven by the ergot group *per se*, but because they induce the activation of 5HT2 receptors [14,124]. Although in the parkinsonian heart, noradrenergic denervation is well stablished, it is known to have an overexpression of adrenergic 5HT2 type receptors.

The significant cardiac effects produced by ergot agonists motivated the use of nonergot DA agonists (e.g., pramipexole (DrugBank ID: DB00413), rotigotine (DrugBank ID: DB05271) or ropinirole (DrugBank ID: DB00268)). The most open to debate drug is pramipexole, since diverse evidence has pointed out a higher occurrence of heart failure in PD patients taking this drug [123,125]. The reason for this relationship is unknown, but it is thought to be due to agonism on α-2-adrenergic receptors [126].

Regarding MAO inhibitors, minimal cardiovascular effects have been described after rasagiline (DrugBank ID: DB01367) administration in conscious rats, treated with high doses of L-DOPA without an amino-acid decarboxylase inhibitor. Moreover, rasagiline has no sympathetic effects, whereas selegiline does (DrugBank ID: DB01037) [127]. Thus, selegiline increased NA plasma levels after L-DOPA administration [128]. However, the extrapolation of these finding to parkinsonian patients is limited, considering that in this disease there is a sympathetic denervation [13].

COMT has a direct relationship with L-DOPA metabolism and, in this line, selective inhibitors of this enzyme, such as tolcapone (DrugBank ID: DB00323) and entacapone (DrugBank ID: DB00494), have great potential to be used as an adjuvant treatment to L-DOPA administration [129]. Although further studies are needed to confirm it, it has been suggested that the combined therapy of tolcapone and L-DOPA does not have autonomic effects on the cardiovascular function of PD patients [130], but further studies are necessary to confirm this result.

Anticholinergic medications are used in the management and treatment of a wide range of diseases, among others, PD. Several lines of evidence have demonstrated that cholinesterase inhibitors can improve the computer-based cognitive performance in individuals diagnosed with dementia with Lewy bodies (for review see [131]). In general, cholinesterase inhibitors (rivastigmine (DrugBank ID: DB00989), donepezil (DrugBank ID: DB00843), and galantamine (DrugBank ID: DB00674)) are known to be associated with bradycardia. However, the cholinesterase inhibitor rivastigmine, has been shown to inhibit both acetylcholinesterase and butyrylcholinesterase, causing an overall increase in acetylcholine [132]. Importantly, donepezil was included in the “known-risk” category of the CredibleMeds list in March 2015, which means that it has a demonstrated risk of acquired enlargement of the QT segment and ventricular arrythmia different to bradycardia [133].

Finally, amantadine (DrugBank ID: DB00915) is an antiviral agent used mostly for PD treatment. This drug is a noncompetitive antagonist of the NMDA receptor, which has the effect of enhancing DA release and reducing DA reuptake [134]. Amantadine is known to have a low side effect profile, but its main side effects can be OH and syncope [134].

Since there are studies demonstrating the detrimental effects of antiparkinsonian drugs and others that fail to find them, it is clear that more studies are needed to assess their possible cardiac adverse effects. This dual situation gives light to the thought that the drugs themselves might not be the direct cause of cardiovascular dysfunction in PD. Knowing that the cardiac system is already compromised in the prodromal stages of PD, the use of different treatments might lead to worsen this situation since there is already an impairment. Therefore, the effect of treatments must be considered to occur in the context of a parkinsonian heart and personalized therapy must be supported, together with a cautious monitorization of cardiac function in PD patients.

Interestingly, during the last years it has been demonstrated that heat shock protein 27 (Hsp27, UniProtKB ID: P04792) has a key role in preventing the fibrillary formation of α-synuclein and also exerts cardioprotection. Thus, Hsp27 could be a promising target to design new therapies focused on both motor and non-motor symptoms without cardiac side effects [65].

## 6. Cardiac Alterations in Neurotoxin-Based Models for PD Research

Neurotoxin-based models have provided significant knowledge about the neuropathology of PD, also offering the possibility to test therapeutic agents [135]. Among them, the most common used neurotoxins are 6-hydroxydopamine (6-OHDA) and 1-methyl-4-phenyl-1,2,3,6-tetrahydropyridine (MPTP), mainly used in nonhuman primates and rodents (rats and mice) [136,137,138]. These neurotoxins are structural analogs of dopamine and they have been demonstrated to induce dopaminergic cell degeneration, the harmful-related processes (e.g., oxidative stress, neuroinflammation), as well as motor and non-motor alterations [139]. Due to their specificity to bind monoamine transporters, they seem to be ideal candidates to study cardiac sympathetic loss in PD. In the following sections, the main findings derived from the experimental parkinsonism induced by 6-OHDA and MPTP are collected.

### 6.1. Hearts in the 6-OHDA Model

The compound 6-OHDA is a benzenotriol in which the hydrogens in positions 2, 4 and 5 of the phenyl ring are replaced by hydroxy groups (PubChem CID: 4624). This compound is able to be selectively taken up by adrenergic terminals, leading to NA and DA. At a physiological pH it is rapidly oxidized, inducing the formation of reactive radical species and neural cytotoxicity (PubChem CID: 4624).

As 6-OHDA cannot cross the blood brain barrier, damage in the dopaminergic system using 6-OHDA is caused by intracranial injection, with limited peripheral effects regarding sympathetic innervation [14]. Hence, catecholaminergic toxicity of 6-OHDA outside the central nervous system must be obtained by systemic administration [14]. It is noteworthy that when the 6-OHDA is administered systemically, the immediate cardiac effects are sympathomimetic: increased blood pressure, bradycardia, fractional shortening or elevation of hematocrit levels [140,141]. For this reason, the preferred intoxication regimen is the application of several doses, time-spaced (usually hours) up to a desired total dose, in order to stabilize the sympathomimetic response [14].

**Figure 4:**
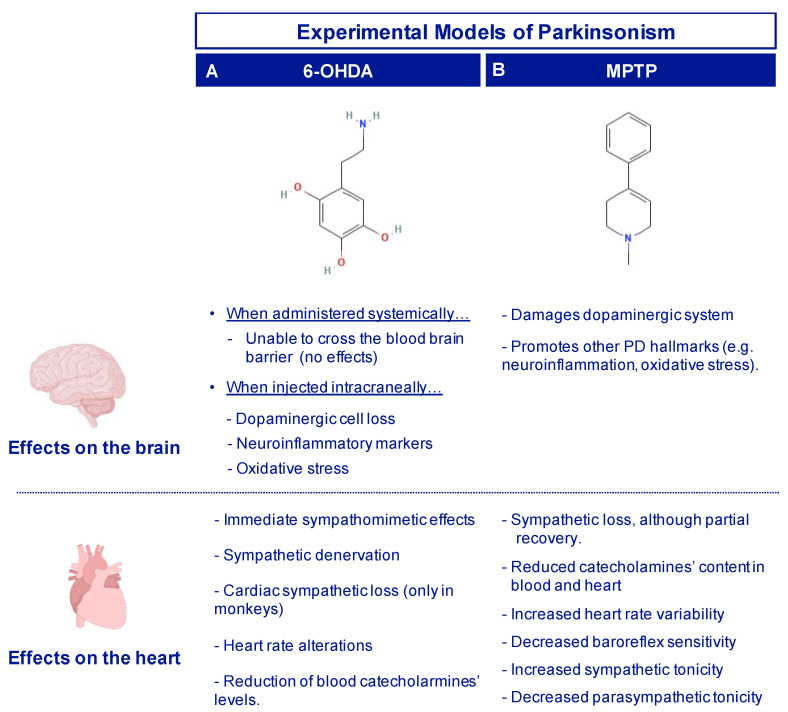
Brain and cardiac changes observed in the toxin-based models for PD research, using 6-OHDA (**A**) and MPTP (**B**).

Cardiac sympathetic loss after systemic administration of 6-OHDA has been shown in cats [142], dogs [140,143], rabbits [144,145], mice [146], rats [147] and nonhuman primates [44,148] (Figure 4A).

Intracranial 6-OHDA infusion in the Substantia Nigra pars compacta (SNpc) of Wistar rats, both uni- and bilaterally, causes cardiac alterations, mainly in the heart rate (especially in the night pattern) and arterial pressure [69,149]. However, signs of sympathetic denervation have not been demonstrated [150] (Figure 4A).

Interestingly, ovarian hormones seem to have a cardiac protective role against intracranial 6-OHDA intoxication in rats [151]. Males bilaterally infused in the SNpc with 6-OHDA showed a decreased mean arterial pressure and heart rate, and increased nitric oxide (NO) levels in the heart and aorta compared to sham animals [69,152]. On the contrary, females did not show changes in those parameters, but when they were subjected to ovariectomy, arterial pressure values, heart rate and NO levels were similar to the males [151].

Recent works on 6-OHDA models have focused on the evaluation of the effect of current human PD therapies, such as L-DOPA and domperidone, showing that both agents promoted alterations in cardiac parameters (e.g., heart beat) [153,154]. In this sense, the use of experimental models are valuable tools to explore the peripheral response to PD treatments and, therefore, to investigate protective alternatives.

Studies of the effects of 6-OHDA on the heart of nonhuman primates are also scarce. Joers and collaborators monitored in vivo cardiac function in rhesus monkeys intoxicated with 6-OHDA (cumulative dose of 50 mg/kg), up to 3 months after the last injection [141]. Circulating levels of catecholamines were significantly reduced after 6-OHDA administration during the whole study and MHED-PET revealed a significant reduction in catecholaminergic innervation, especially in the inferior myocardium of the left ventricle. These in vivo results were subsequently coupled with *postmortem* measurements [148]. Cardiac sympathetic denervation was confirmed by a significant reduction of TH expression in the left ventricle (both in fibers and nerve bundles), which correlated with a reduction in PGP9.5 (a neuronal marker). This loss was greater in the inferior left ventricle wall than in the lateral wall, similarly to the cardiac denervation patterns observed in humans [33].

In summary, the available works in the 6-OHDA model have shown that its systemic administration is able to cause sympathetic postganglionic degeneration and a reduction of catecholaminergic fibers in the heart, similar to that which is observed in PD patients [44,69] (Figure 4A). However, even if systemic 6-OHDA administration induces cardiac dysautonomia in nonhuman primates, it must be considered that to obtain a complete PD-like state, this model must be complemented with other tools that would cause dopaminergic cell loss in the nigrostriatal system.

### 6.2. Hearts in the MPTP Model

MPTP is a tetrahydropyridine (a member of the family of methylpyridines), in which the base chemical structure is a 1,2,3,6-tetrahydropyridine substituted by a methyl group at position 1 and a phenyl group at position 4 (1-methyl-4-phenyl-1,2,3,6-tetrahydropyridine) (PubChem CID: 1388). MPTP is a dopaminergic proneurotoxin, which can cross the blood brain barrier and be transformed to its active toxic form MPP+ by the astrocytic MAO-B enzyme. This compound is then taken up by dopaminergic transporters, inducing irreversible chemical, pathological and clinical changes similar to those found in PD.

Since it is a liposoluble proneurotoxin, MPTP can be administered systemically and exert both peripheral and central nervous system effects by crossing the blood brain barrier [155]. However, like 6-OHDA, the cardiac effects of MPTP are highly dependent on the dose, administration regimen and species.

Under MPTP intoxication, alterations in the cardiac sympathetic system have been demonstrated in mice [156,157], rats [158] and nonhuman primates [159]. Some authors have shown that, even if cardiac sympathetic loss was persistent (i.e., cardiac NA and dopamine loss), a partial recovery was detected [158,160,161,162]. Together with sympathetic autonomic dysfunction, impaired autonomic function has been detected in the hearts of MPTP-treated mice, including increased heart rate variability, lower baroreflex sensitivity, increased sympathetic and decreased parasympathetic tonicities [163] (Figure 4B).

A longitudinal study in MPTP-intoxicated monkeys showed that the cardiac effect varies over the time [161]. Immediately after intoxication (acute phase), catecholamine content in the blood is reduced due to preganglionic damage. After months of repeated MPTP administration (subacute phase), levels of catecholamines are reduced both in the plasma and in the heart. Finally, in a long-term phase after intoxication, a recovery is experienced, where catecholaminergic levels return to normal levels and there is no evidence of cardiac nerve loss. A recent characterization of the subacute phase, confirmed the lack of axonal loss in the heart of parkinsonian monkeys, but significantly reduced TH immunoreactivity in the cardiac nerve fascicles compared with the control animals was found, together with the presence of α-synuclein deposits in the left ventricle [159]. These results agree with a previous study in mice, where despite cardiac sympathetic dysfunction, cardiac fibers were preserved [162]. Interestingly, a significant increase in the expression of vesicular monoamine transporter 2 (VMAT2) and NA transporter (NET) were detected in the nerve fascicles of the heart of MPTP-intoxicated monkeys, suggesting the existence of compensatory mechanisms [159]. Altogether, these findings point out that in the early stages of PD, the degeneration of the TH+ fibers in the heart might precede the axonal loss that has been confirmed in patients. In addition to these changes, intoxication with MPTP also induces a reduced NA content and cardiac uptake of MIBG, together with a decreased NET density in the postganglionic nerves [158,164]. Recent studies from our group have shown that in the hearts of MPTP-intoxicated monkeys there was a decrease in NA turnover together with an increase of normetanephrine (NMN), a peripheral metabolite of NA [118].

Recently, it has been described that, together with the loss of dopaminergic neurons in the SNpc, MPTP intoxication can induce an important decrease of TH+ cells in the LC, RVLM, and NTS [163]. These findings are associated with a significant reduction in dopamine, NA, and adrenaline levels in the abovementioned nuclei, thus confirming the degeneration of sympathetic pathways [163]. The loss of dopaminergic neurons in the brainstem produced by MPTP induces alterations in autonomic cardiovascular function. Additionally, recent results from our laboratory have demonstrated monkeys intoxicated with MPTP showed a reduction in total TH expression in both cardiac ventricles, but especially in the left one, together with a significant increase in phosphorylated TH. In addition, our data demonstrated a significant correlation between total TH levels in the heart tissues and the number of TH + neurons in SNpc [165]. These results support the key interrelationship between brain and heart and the need to design new therapeutical strategies in order to reduce or eliminate the brain and cardiac alterations found in PD.

Given the contribution of α-synuclein to PD pathology, Cano-Jaimez and collaborators explored the role of this protein in the cardiac NA content [166]. In contrast to dopaminergic neurons, elimination of α-synuclein did not affect the detrimental effect of MPTP in the heart. Therefore, these results suggest differential brain and cardiac susceptibility to MPTP. Importantly, cholinergic innervation in the heart of MPTP models is not altered, resembling the condition observed in PD patients [52,159].

It seems that the use of MPTP creates some controversies regarding the study of cardiac alterations. Firstly, there is the evidence of recovery mechanisms, which might be avoided by considering different end-point times. Secondly, even though cardiac sympathetic loss is detected, there is no evidence of cardiac denervation. Some authors consider that this aspect can contribute to understanding the autonomic changes in the early phases of PD [159], while others argue that the neurodegenerative process seen in human PD has a slower progression compared to the one induced by MPTP intoxication (Figure 4B). Therefore, chronic MPTP administration regimens should be analyzed in terms of cardiac dysautonomia. In addition, apart from the SNpc, MPTP also damages TH+ sympathetic nuclei (such as the locus coeruleus, RVLM and NTS), reducing the number of noradrenergic cells and the blood levels of catecholamines [163]. Thus, cardiac sympathetic loss might be a consequence of the preganglionic lesion, the opposite to what has been described in PD patients, in which sympathetic denervation seems to be specific to the heart and plasma catecholamines levels are not altered [13,31].

## 7. Conclusions

PD is a neurodegenerative disorder that goes beyond dopaminergic neuronal death and the occurrence of Lewy bodies, since numerous studies have shown that multiple systems are affected. Among them, cardiovascular alterations are one of the most relevant comorbidities in PD. In particular, it has been shown that PD patients show a loss of post-ganglionic sympathetic innervation in the heart, together with functional and molecular changes. However, many questions are still unsolved. Future lines of research might focus on the mechanisms involved in cardiac dysfunction in PD, in which PD-related genes could play a key role. In addition, noncoding RNAs could also provide some clues to understand (and ideally modulate) the brain–heart axis. The use of experimental models is key to understanding the pathological mechanisms involved, as well as designing therapies to treat the neurodegenerative process and the associated clinical manifestations, such as cardiovascular alterations.

## Data Availability

Not applicable.
